# Case report: A case of delayed cutaneous metastases from signet-ring cell mixed-type gastric cancer

**DOI:** 10.3389/fonc.2023.1105080

**Published:** 2023-02-27

**Authors:** Shaohua Yao, Peng Zhou, Yiqing Li, Qin Li

**Affiliations:** ^1^ Department of Gastrointestinal Surgery, Huazhong University of Science and Technology Union Shenzhen Hospital, Shenzhen, China; ^2^ Department of Vascular Surgery, Union Hospital, Tongji Medical College, Huazhong University of Science and Technology, Wuhan, China

**Keywords:** signet-ring cell gastric carcinoma, cutaneous metastases, late recurrence, case report, post operative metastasis

## Abstract

**Background:**

Signet-ring cell gastric carcinoma is a highly malignant tumor, with the characteristics of strong invasiveness, rapid progression, a high degree of malignancy, and generally poor prognosis. The most common site of metastases is the abdominal organs, especially the liver, while delayed cutaneous metastases are rare.

**Case presentation:**

We report a case of cutaneous metastases on the head, groin, and thigh, which recurred 7 years after signet-ring cell gastric carcinoma surgery. The patient was diagnosed with a 2.0×1.5×1.0cm tumor at the angle of stomach, and treated with Billroth II distal gastrectomy accompanied with D2 lymph node dissection. According the pathology, the stage was pT1N3M0. Then the patient received two cycles of oxaliplatin and tegafur chemotherapy, which was discontinued due to the inability to tolerate the side effects of chemotherapy. Seven years after the surgery, the patient initially presented with a fleshy mass on the head and beaded nodules in the groin; then, the mass gradually became larger, along with the thighs turning red, swollen, and crusty. Firstly, the patient was diagnosed with “lower extremity lymphangitis” and treated mostly with anti-inflammatory, promote lymphatic return, detumescence and elastic force cannula in vascular surgery department. However, the symptoms relieved insufficient. Finally, the skin biopsy indicates a signet-ring cell gastric carcinoma cutaneous metastasis. The whole-body PET-CT examination showed multiple nodules with increased metabolism. Then the patient was transferred to The Department of Oncology for further chemotherapy.

**Conclusion:**

Our case highlights that gastric tumor recurrence and metastasis should be highly suspected when skin lesions appear in patients with signet-ring cell gastric carcinoma. At the same time, multidisciplinary consultation and close cooperation between surgeons, oncologists, and dermatologists are of great significance to the diagnosis and treatment of this disease.

## Introduction

Cutaneous metastases of gastric carcinoma are extremely rare. Multiple studies have reported the prevalence of cutaneous metastases ranging from 0.2% to 2.2% (1), predominantly in males. The outcomes of postoperative gastric carcinoma are usually assessed by the 5-year survival rate. Cutaneous metastases generally occur within 1-3 years postoperatively, whereas reports of cutaneous metastases over 5 years are even rarer. Among histopathological subtypes of gastric carcinoma, signet-ring cell (SRC) carcinoma has a greater propensity for distant and cutaneous metastases. Furthermore, SRC gastric carcinoma is more prone to lymphatic metastasis compared with other histopathological subtypes of gastric carcinoma (2).

## Case report

**Figure 1:**
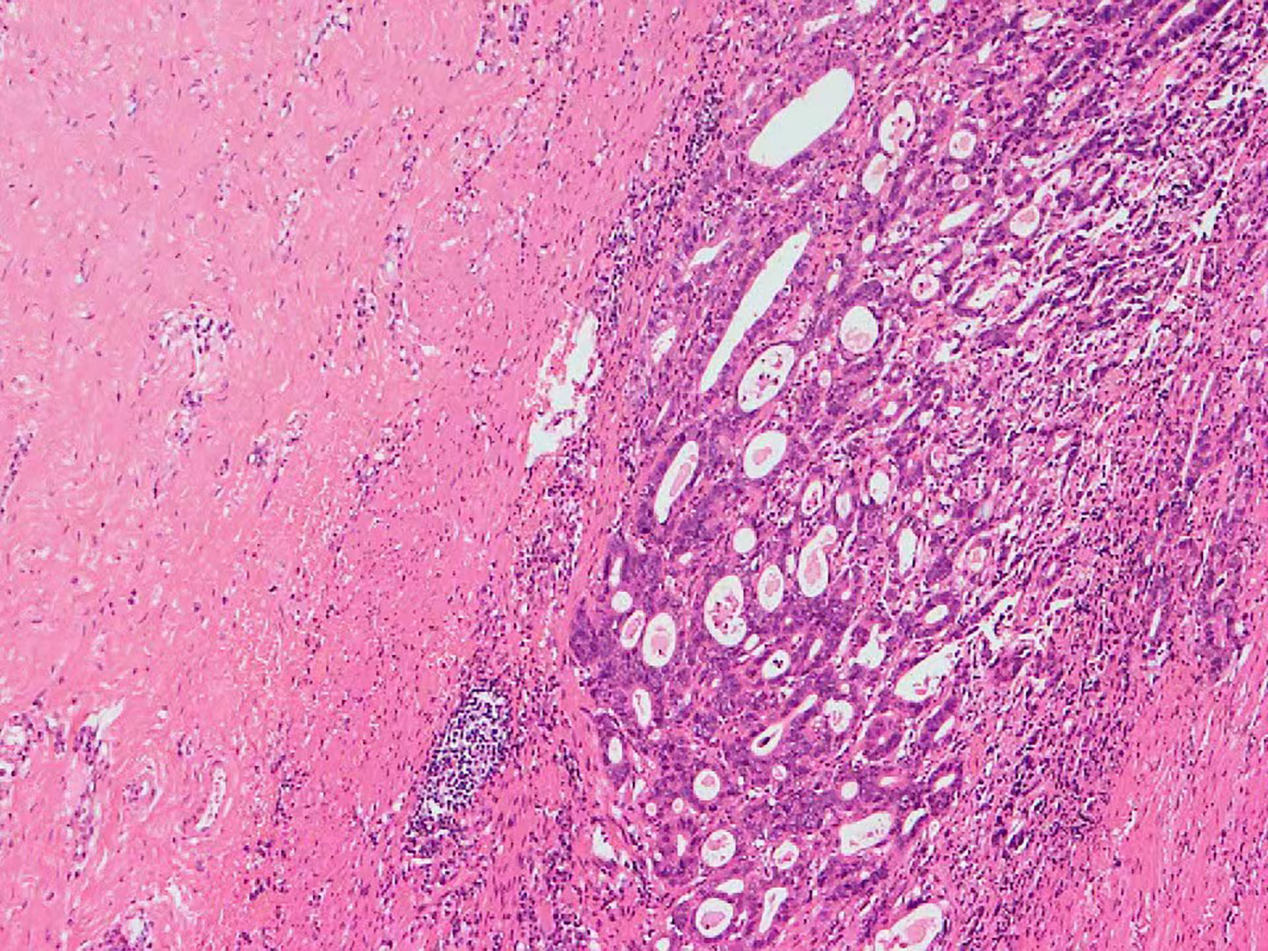
Postoperative pathology of gastric carcinoma in 2015.

A 61-year-old man was admitted to our hospital in May 2022 for three progressively enlarging masses in the left groin area and head, along with redness and swelling of the left thigh for more than half a year. He had a history of a major gastrectomy in 2015 after being diagnosed with gastric carcinoma. The patient was diagnosed with a 2.0×1.5×1.0cm tumor at the angle of stomach, and treated with Billroth II distal gastrectomy accompanied with D2 lymph node dissection. The postoperative histopathology of the primary lesion was mixed invasive adenocarcinoma, of which signet-ring cell carcinoma accounted for 80%, and tubular adenocarcinoma accounted for 20% (**Figure 1**). The pathological stage was pT1N3M0. So, the patient received two cycles of oxaliplatin and tegafur chemotherapy, which was discontinued due to the inability to tolerate the side effects of chemotherapy. No tumor recurrence was found in the annual gastroscopy and CT-scan for 4 years after the operation; then, no follow-up was performed due to the COVID-19 epidemic.

However, after 6 years, the patient developed a beaded nodular tumor in the left groin area and two fleshy nodules on the scalp, accompanied by redness and swelling from the upper left thigh to the groin area; there was no obvious fever or pain. This patient was diagnosed with “lymphangitis of lower limbs” in several major hospitals and was prescribed anti-inflammatory and detumescence treatment. After repeated treatment, the tumor in the left groin area and scalp gradually increased, and the skin of the left lower extremity was red, swollen, and scleroderma-like. Finally, he came to our hospital for treatment.

**Figure 2:**
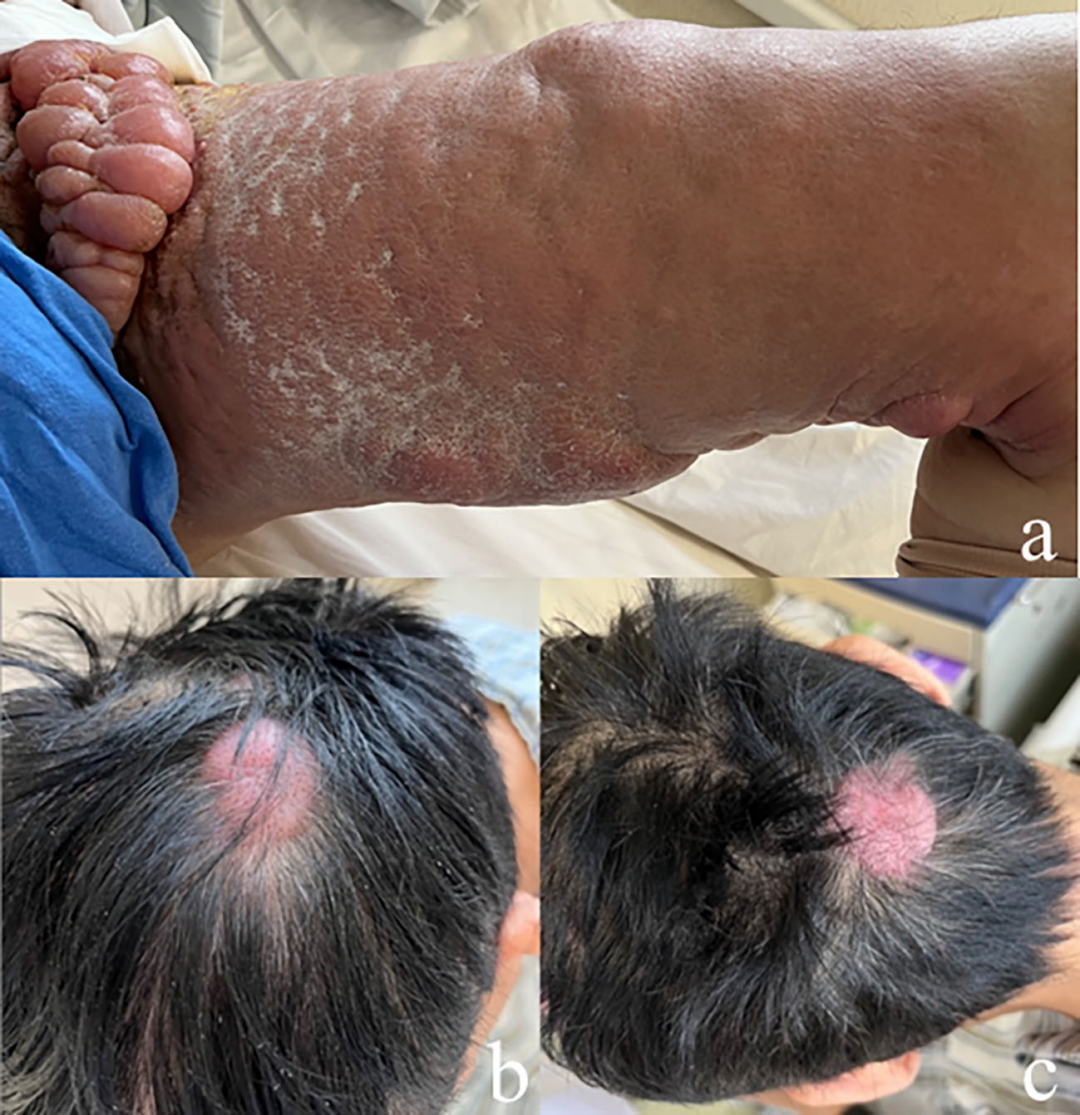
Cutaneous metastatic carcinomas. **(A)** Beaded mass in left groin area and erythematous scleroderma of the left thigh; **(B, C)** Two fleshy masses on the scalp.

**Figure 3:**
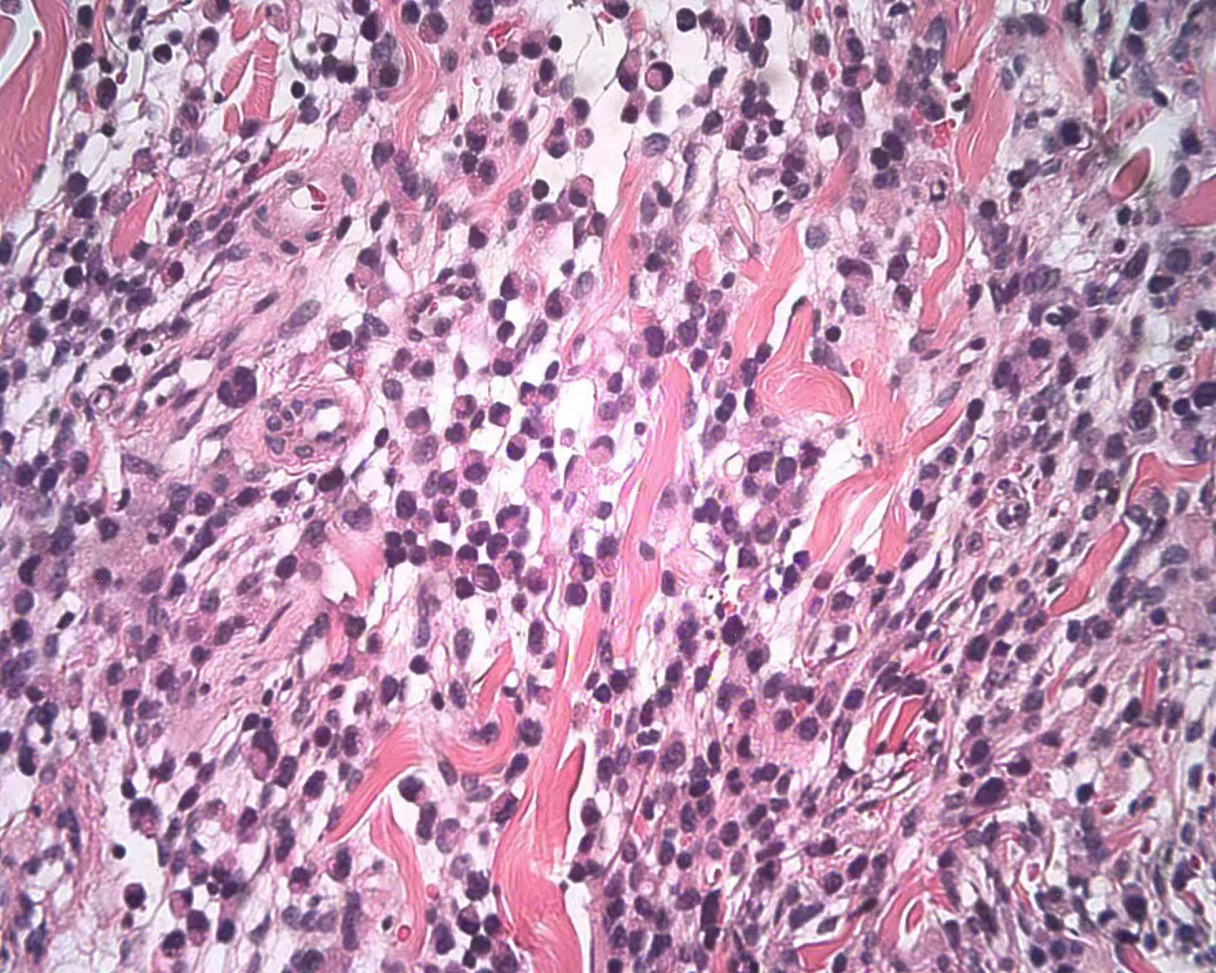
Skin biopsy of the groin mass; the pathology showed metastatic poorly differentiated carcinoma, some of which were signet-ring cell carcinoma.

**Figure 4:**
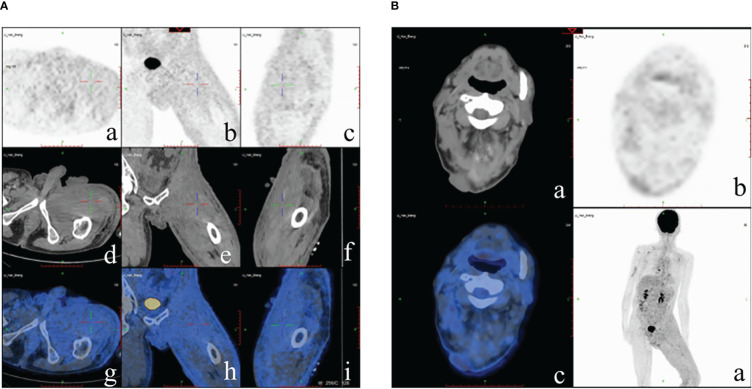
PET-CT examination. **(A)** Multiple humus-like hypodense nodules on the skin surface from the left groin to the thigh, secondary lymphedema, and slightly increased metabolic diffusion; **(B)** Localized thickening of the skin on the top of the left head and back of the neck and slightly increased metabolism.

A physical examination revealed moderate pitting edema of the left lower extremity, redness and swelling of the thigh with scleroderma-like changes, bead-like nodules of about 5*20cm in the groin area (**Figure 2A**), and two fleshy masses of about 3*3cm in size on the scalp (**Figures 2B, C**). The laboratory examinations showed that the blood routine, procalcitonin, liver, and kidney function were not abnormal, and the tumor markers carbohydrate antigen 72-4 and CA125 were elevated. A skin biopsy was performed on the groin mass, and the pathology showed metastatic poorly differentiated carcinoma, some of which were signet-ring cell carcinoma; immunohistochemical staining showed: PCK(+), CK7(+), CK20(-), CDX2(+), SATB2(-), and HER2(0) (**Figure 3**). A whole-body PET-CT examination showed multiple humus-like hypodense nodules on the skin surface from the left groin to the thigh, secondary lymphedema, and slightly increased metabolic diffusion (**Figure 4A**). There was also localized thickening of the skin on the top of the left head and back of the neck, and slightly increased metabolism. The above suggested the possibility of malignant tumor metastatic lesions (**Figure 4B**). After the diagnosis was confirmed, the patient was transferred to the oncology department for chemotherapy of Nivolumab combined with SOX. Four times of chemotherapy had been performed, and the efficacy was evaluated as reduced SD. Due to the COVID-2019 epidemic, the patient did not come to our hospital for subsequent chemotherapy treatment as planned.

## Discussion

Cutaneous metastases usually develop from breast cancer, lung cancer, colorectal cancer, ovarian cancer, and other tumors (3), while metastases from stomach cancer to skin are extremely rare. Cutaneous metastases of gastric carcinoma are classified into the following three categories: (a) nodular, (b) inflammatory, and (c) sclerodermoid; the most common type is nodular type, followed by inflammatory (4). However, these three types occurred simultaneously in this case. The most common sites of metastases include the neck, back, abdomen, and inguinal regions. Meanwhile, the lesions can evolve into single or multiple nodules with an erysipelas-like morphology (also confirmed in this patient). Erysipelas carcinoma resembles an acute skin infection; different from skin infections, erysipelas carcinoma does not cause fever or leukocytosis, and antibiotics are ineffective (5). This case presented with a nodular appearance at first, followed by erysipelas-like changes and skin scleroderma-like changes in the lower extremities. After being treated as lymphangitis and lymphedema of the lower limbs in many large hospitals in China, his symptoms were not alleviated. The diagnosis was not confirmed until a skin biopsy was performed half a year later; so, the best treatment opportunity was missed.

The pathogenesis of cutaneous metastases is still unclear. According to previous observations, poorly differentiated adenocarcinomas with signet-ring cell characteristics are closely related to the occurrence of cutaneous metastases. The proportion of SRC is inversely related to aggressive behavior, higher risk of metastases and poor prognosis in mixed-type gastric cancer (6). Some of the potential mechanisms are hematogenous or lymphatic metastasis. Holmgren L proposed the concept of “tumor dormancy” (7), as the reason for delayed skin metastases in gastric cancer, which refers to the state of small residual lesions or isolated micrometastases without causing symptoms for a long period of time (8). However, the triggers that activate dormant cells to lead to relapse have not been identified. In this case, the patient showed advanced recurrence of stage IIB gastric cancer, histopathology of the primary tumor was pT1 and N3, and the patient received only two adjuvant chemotherapy sessions after surgery. If an adequate course of adjuvant chemotherapy is given, it may reduce the risk of cancer recurrence.

Cutaneous metastasis of gastric cancer is one of the markers of an advanced tumor stage, suggesting a poor prognosis for patients, but about 61% of patients still receive active treatment with surgery, chemotherapy, or radiation therapy (9). Although the application of active treatment can significantly improve prognosis, there are many patients did not tolerate chemotherapy which likely increased their recurrence risk of disease. A new study analyzed the molecular profiling of signet-ring cell carcinoma (SRCC) from the stomach and colon by using NGS, immunohistochemistry and *in situ* hybridization, suggest that SRCCS harbor a similar molecular profile, regardless of the tumor location, means tailored therapy may become available for these patients in the future (10). Liu Shuzhen et al. reported 51 cases of skin metastases of malignant tumors; of these, 8 patients did not receive active antitumor treatment and died within 4 months (11). Among the 43 patients who received treatment, the median survival time was significantly longer than untreated patients, indicating that active treatment can prolong the survival of patients with skin metastases of malignant tumors, especially those without vital organ metastases.

Consequently, Signet-ring cell gastric cancer has a higher incidence of long-term cutaneous metastasis than other types of gastric cancer, and the possibility of tumor recurrence and metastasis should be highly suspected when skin lesions appear in patients with a clinical history of gastric cancer. Therefore, early diagnosis and treatment is extremely important. At the same time, multidisciplinary consultation and close cooperation between surgeons, oncologists, and dermatologists are of great significance to the diagnosis and treatment of this disease.

## Data availability statement

The raw data supporting the conclusions of this article will be made available by the authors, without undue reservation.

## Ethics statement

The studies involving human participants were reviewed and approved by Ethics Committee of Wuhan Union Hospital, Tongji Medical College, Huazhong University of Science and Technology, Wuhan, China. The patients/participants provided their written informed consent to participate in this study. Written informed consent was obtained from the patient for the publication of the case report and the accompanying images.

## Author contributions

QL and YL made substantial contributions to design the research work. SY and PZ made substantial contributions to collecting the clinical data. SY and PZ wrote the initial draft of the manuscript. QL and YL revised the paper for important intellectual content. All authors contributed to the article and approved the submitted version.
